# Perspectives and Awareness of Environmental Sustainability in the Infection Prevention and Control Community Nationally

**DOI:** 10.1017/ash.2024.130

**Published:** 2024-09-16

**Authors:** Abarna Pearl, Dana Pepe, Preeti Mehrotra

**Affiliations:** Beth Israel Deaconess Medical Center

## Abstract

**Background:** Healthcare contributes significantly to waste production and greenhouse gas emissions. This became especially apparent during the COVID-19 pandemic. Yet there is modest recognition of this issue, particularly within decision-making in Infection Prevention and Control (IPC). The aim of our study was to gauge general knowledge and attitudes of hospital epidemiologists (HEs) and infection preventionists (IPs) around the intersection of environmental sustainability and IPC, and to identify related institutional practices. **Methods:** An online survey, composed of ten questions related to environmental sustainability in IPC, was created and emailed to members of the SHEA Research Network (SRN), a national consortium of healthcare facilities collaborating on IPC research, from August - October 2023. Survey answers were collated via Redcap© and descriptive results were obtained. **Results:** Forty-two individuals (33 HEs, 7 directors of IPC, and 2 IPs) from unique institutions completed the survey. Thirty (71.4%) were from academic medical centers, 5 (11.9%) were from VA medical centers and 7 (16.7%) were from community hospitals. Over half of participants correctly estimated the amount of waste and carbon emissions produced annually by the US healthcare system (6 million tons and 8.5% of national emissions, respectively). However, only 42.9% considered environmental sustainability concerns important or very important when making IPC decisions. Fifteen (34.9%) had an environmental sustainability committee at their institution and of these, 8 had an established relationship with the IPC department. The most common techniques to promote sustainability amongst institutions were water/energy conservation (59.5%), reusable personal protective equipment (52.4%) and Leadership in Energy and Environmental Design (LEED) certification (47.6%). When asked which efforts they would support at their institution, 28.6% would eliminate the use of single-use endoscopes and one third would avoid use of ethylene oxide for sterilization. In deciding whether to support environmental sustainability measures, key considerations participants articulated were patient safety concerns, knowledge about effectiveness and costs, and administrative support. **Conclusion:** Although there is growing awareness around the contribution of the healthcare industry to carbon emissions and waste production, IPC professionals have not yet universally adopted measures to promote environmental sustainability. In our survey, many participants acknowledged the importance of balancing patient safety and sustainability concerns. Our study demonstrates the need for more research and education to inform decisions around environmentally sustainable efforts in IPC that also preserve patient safety. Additionally, professional and regulatory bodies must acknowledge and promote the importance of environmental sustainability in IPC decision-making.

